# Récidive d'un meningiome géant agressif à développement intra et extra-cranien

**DOI:** 10.11604/pamj.2013.16.123.3198

**Published:** 2013-11-29

**Authors:** Aliou Amadou Dia

**Affiliations:** 1 Service de radiologie de l'hôpital Saint-Jean de Dieu, Thiès, Sénégal

**Keywords:** Meningiome, processus intra-cranien, tuméfaction, meningioma, intracranial process, swelling

## Image en médicine

Nous rapportons un cas de reprise évolutive méningiome agressif ostéolytique, à développement intra et extra-crânien chez un patient de 37 ans qui présente depuis 10 ans une volumineuse voussure de la région pariéto-occipitale. Le patient a bénéficié en 2010 d'une exérèse partielle d'un processus expansif intracrânien qui s'est révélé être un méningiome méningothélial à l'anatomopathologie. Le patient consulta de nouveau en juillet 2013 pour un syndrome d'hypertension intra-crânien associée à une hémianopsie latérale homonyme et une hémiparésie droite évoluant en tache d'huile. L'examen physique montrait une volumineuse tuméfaction pariéto-occipitale en dôme non douloureuse à la palpation, de consistance ferme et qui ne cesse d’évoluer depuis l'intervention chirurgicale, selon le patient. La tomodensitométrie montrait un volumineux processus expansif intracrânien pariéto-occipital gauche, extra-axial, mesurant 11.5 cm x 10.5 cm dans le plan axial, ostéolytique avec une extériorisation d'une partie du processus tumoral en dehors de la boite crânienne. Cette masse tumorale se rehaussait de façon intense après injection et présentait une densité hétérogène par la présence de plages kystiques. Sur le scout view et les fenêtres osseuses, on notait une ostéolyse de la voûte, hérissée de spicules osseux en « rayon de soleil » et des calcifications intra-tumorales. On notait une vaste plage d'oedème péri-lésionnel, un important effet de masse sur la corne occipitale ventriculaire gauche et la ligne médiane avec un engagement sous-falcoriel et temporal gauche ainsi qu'une discrète hydrocéphalie d'exclusion. L'aspect était évocateur d'une reprise évolutive d'un volumineux méningiome pariéto-occipital gauche agressif.

**Figure 1:**
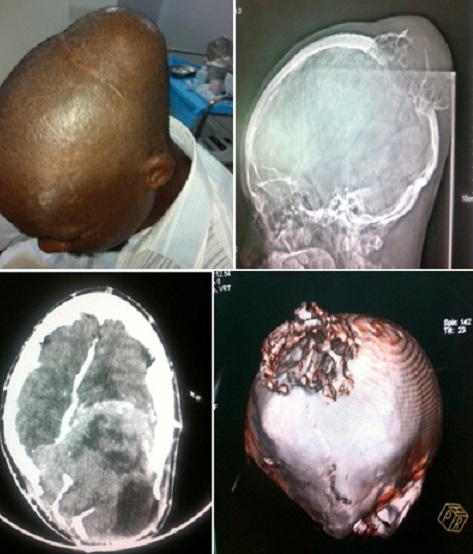
A) Volumineuse tuméfaction pariéto-occipitale en “dôme” associée à une cicatrice de craniotomie en avant de la tumeur; B) Scout View montrant une importante ostéolyse de la voûte, hérissée de spicules osseux en « rayon de soleil » et des calcifications intra-tumorales.

